# Supplementary material for: Using Dual Toll-like Receptor Agonism to Drive Th1-Biased Response in a Squalene- and α-Tocopherol-Containing Emulsion for a More Effective SARS-CoV-2 Vaccine
Source: Pharmaceutics. 2022 Jul 12;14(7):1455. doi: 10.3390/pharmaceutics14071455 (PMC9318334; doi:10.3390/pharmaceutics14071455)
Supplement: Supplementary file 1 [file pharmaceutics-14-01455-s001.zip › pharmaceutics-1779935-supplementary.pdf]

Supplementary Materials

Table S1: *In vivo* study design for AddaS03-like emulsions loaded with INI-4001 and INI-2002, singly or in combination. AddaS03-like emulsions with and without TLRs were formulated in DPBS, pH 6.8 and MF59-like emulsion was formulated in 10 mM citrate, pH 6.0. RBD protein was admixed into the formulation before immunization. Eight C57BL/6 mice were used per group, except five mice were used for the naïve group. In order to compensate for the adjuvant effect of squalene, the total amount of squalene was measured by RP-HPLC, and the squalene doses were equalized using the AddaS03-like vehicle formulation. All emulsions were prepared separately.

Group	RBD (µg)	INI-4001 dose (nmol)	INI-2002 dose (nmol)	Actual Molar Ratio (4001/2002)	Squalene dose (µg)
Naïve	-	-	-	-	-
Antigen only	1	-	-	-	-
AddaS03-like	1	-	-	-	144
AddaS03-like + 4001	1	0.876	-	-	144
	1	8.76	-	-	186
AddaS03-like + 2002	1	-	0.876	-	144
	1	-	8.76	-	144
AddaS03-like + 4001:2002	1	8.76	0.876	NA	144
	1	8.76	8.76	1.03	144
MF59-like	1	-	-	-	144

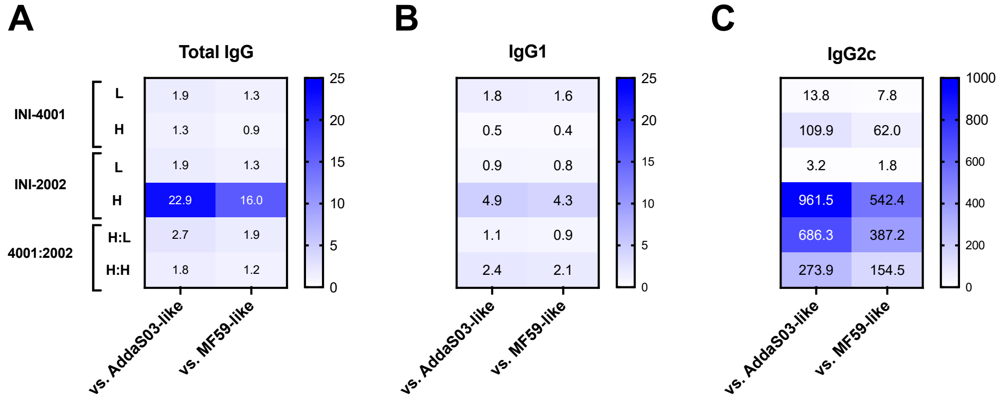

Figure S1: Fold-change in RBD-specific IgG titers from serum. Values are presented as fold-change of mean RBD-specific antibody titers for each group over AddaS03-like emulsions or MF59-like emulsions for (**A**) total IgG, (**B**) IgG1, and (**C**) IgG2c. Antibody titers were measured from the serum of C57BL/6 mice fourteen days post-secondary immunization with 1 µg SARS-CoV-2 RBD antigen admixed with emulsion formulations. Emulsion doses contained 8.76 nmol (10 µg) or 0.876 nmol (1.0 µg) INI-4001, and 8.76 nmol or 0.876 nmol INI-2002 separately or co-encapsulated with equal amounts of squalene. Dose values on the graphs are given as low dose, (L, 0.876 nmol) or high dose (H, 8.76 nmol).

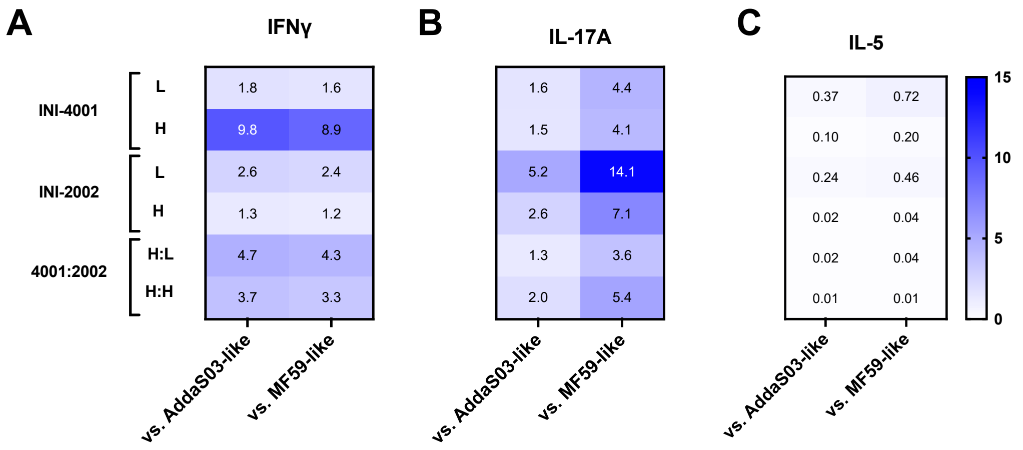

Figure S2: Fold-change in RBD-specific cytokine production in cells isolated from the draining lymph node (DLN). Values are presented as fold-change of mean RBD-specific cytokine amount for each group over the mean of AddaS03-like emulsions or MF59-like emulsions for (**A**) IFNγ, (**B**) IL-17A, and (**C**) IL-5. Mice were immunized with 1 µg SARS-CoV-2 RBD antigen admixed with emulsions. Emulsion doses contained 8.76 nmol (10 µg) or 0.876 nmol (1.0 µg) INI-4001, 8.76 nmol or 0.876 nmol INI-2002 separately or co-encapsulated with equal amounts of squalene. Dose values on the graphs are given as low dose, (L, 0.876 nmol) or high dose (H, 8.76 nmol) for INI-4001 and/or INI-2002.
